# Non-steroidal anti-inflammatory drugs use in older adults decreases risk of Alzheimer’s disease mortality

**DOI:** 10.1371/journal.pone.0222505

**Published:** 2019-09-17

**Authors:** Julián Benito-León, Israel Contador, Saturio Vega, Alberto Villarejo-Galende, Félix Bermejo-Pareja

**Affiliations:** 1 Department of Neurology, University Hospital “12 de Octubre”, Madrid, Spain; 2 Network Center for Biomedical Research in Neurodegenerative Diseases (CIBERNED), Madrid, Spain; 3 Department of Medicine, Complutense University, Madrid, Spain; 4 Department of Basic Psychology, Psychobiology and Methodology of Behavioural Sciences, University of Salamanca, Salamanca, Spain; 5 Arévalo Health Center, Arévalo, Ávila, Spain; 6 Clinical Research Unit (I+12), University Hospital “12 de Octubre”, Madrid, Spain; University of Malaya, MALAYSIA

## Abstract

Alzheimer disease (AD) mortality risk in a large cohort of subjects treated or not with non-steroidal anti-inflammatory drugs (NSAIDs) is unknown. Our objective was to determine whether NSAIDs use is associated with decreased risk of AD mortality. In this prospective, population-based study (Neurological Disorders in Central Spain [NEDICES]) of 5,072 people without AD (aged 65 years and older), sociodemographic, comorbidity factors, and current medications were recorded at baseline. Community-dwelling older adults were followed for a median of 12.7 years, after which the death certificates of deceased participants were examined. 2,672 (52.7%) of 5,072 participants died, including 504 (18.9%) NSAIDs users and 2,168 (81.1%) non-users. Of the 2,672 deceased participants, 113 (4.2%) had AD as a cause of death (8 [1.6%] among NSAIDs users and 105 [4.8%] among non-users, chi-square = 10.70, p = 0.001). In an unadjusted Cox model, risk of AD mortality was decreased in NSAIDs users (hazard ratio [HR] for AD mortality = 0.35, 95% confidence interval [CI] 0.17–0.72, p = 0.004) when compared to non-users. After adjusting for numerous demographic factors and co-morbidities, the HR for AD mortality in NSAIDs users was 0.29, 95% CI 0.12–0.73, p = 0.009. Stratified analyses showed a significantly decreased risk of AD mortality with aspirin, whereas non-aspirin NSAIDs only showed a statistical trend toward significance in the adjusted Cox regression models. NSAIDs use was associated with 71% decreased risk of AD mortality in older adults. Our results support the hypothesis that NSAIDs use is a protective factor of developing AD.

## Introduction

The development and evaluation of new therapies to slow the progression of Alzheimer’s disease (AD) is a public health priority. Currently, it is an exciting time as some traditional drugs, such as non-steroidal anti-inflammatory drugs (NSAIDs), and many others in development might have an impact in reducing the risk of AD.[1] In 1990, it was reported for the first time that patients with rheumatoid arthritis receiving NSAIDs showed a markedly reduced prevalence of AD compared to the overall population.[2] From then, different independent observational studies in humans have found that NSAIDs use is associated with a decreased risk of developing AD.[3] However, clinical trials, mostly using selective cyclooxygenase-2 inhibitors, have so far shown a null efficacy for AD treatment.[4] In fact, recently in a 2-year double-masked pharmaco-prevention trial, enrolling 195 AD family history-positive elderly, sustained treatment with naproxen sodium 220 mg twice daily did not reduce apparent progression of presymptomatic AD.[5] Notwithstanding, interest in NSAIDs has been sparked in AD research by the notion that these medications might have a mode of action beyond inflammation; specifically, their neuroprotective effects might be also mediated by alteration of oxidative phosphorylation and possibly the ribosome pathway,[6] as well as inhibition of mitochondrial Ca2+ overload.[7]. Oxidative stress has long been considered as a component of the many pathophysiological events of AD.[8]

Prospective population- or community-based based cohort studies or community-based cohort studies are preferable for studying whether NSAIDs use offer some protection against the development of AD because they decrease sources of bias and confounding.[9] To date, twelve population- or community-based studies have examined the risk of AD among those who take NSAIDs.[10] These studies have been included in a recent meta-analysis that concluded that NSAIDs use was significantly associated with reduced risk of AD compared with those who did not use NSAIDs. This association existed in studies including all NSAID types, but not in aspirin, acetaminophen or non-aspirin NSAIDs.[10] However, most these studies were not adjusted for arterial hypertension, an important variable, since this condition is associated with both NSAIDs use and AD.[10–12]

Surprisingly, AD mortality risk in a large cohort of participants treated or not with non-steroidal anti-inflammatory drugs (NSAIDs) is unknown. Another currently unexplored method to assess this potential relationship is to study NSAIDs use among deaths for which AD have been assigned as a contributory cause (here after referred to as “AD mortality”).

We aimed to reevaluate the existing uncertainty regarding the effects of NSAIDs use on risk of AD by examining whether AD mortality is decreased in NSAIDs users compared to non-users. Towards this purpose, we used data from the Neurological Disorders in Central Spain (NEDICES) study, a prospective population-based study, in which participants were followed for a median of 12.7 years, after which the death certificates of deceased participants were examined. Our statistical analyses were adjusted for several confounders, including arterial hypertension. We also performed additional analyses stratified by individual NSAID type (i.e., aspirin vs non-aspirin NSAIDs).

## Material and methods

### Statement of ethics

All the participants included in the study gave their written informed consent after full explanation of the procedure. The study was approved by the ethical standards committee on human experimentation at the University Hospitals “12 de Octubre” (Madrid) and “La Princesa” (Madrid).

### Study population

Data for these analyses were derived from the NEDICES study, a longitudinal, population-based survey of the prevalence, incidence, mortality, and determinants of major age-associated conditions of the elderly, including Parkinson’s disease, essential tremor, stroke, and dementia.[13–18] Detailed accounts of the NEDICES study population and sampling methods have been published.[19–21] The survey area consisted of three communities: (1) Margaritas (approximately 14,800 inhabitants), a working-class neighborhood in Getafe (Greater Madrid); (2) Lista (approximately 150,000 inhabitants), a professional-class neighborhood in the Salamanca district (Central Madrid), and (3) Arévalo (approximately 9,000 inhabitants), the agricultural zone of Arévalo County (125 km northwest of Madrid).

### Study evaluation

Briefly, at the time of their baseline assessment (1994 to 1995), participants were interviewed face-to-face using a screening questionnaire to collect data on demographics, current medical conditions, smoking (ever vs. never), and drinker (ever/at least once per week vs. never). At this time, participants were asked to bring all medications taken in the past week to the clinic, where the interviewer recorded the name of each one. As in prior studies,[22, 23] participants were also asked to rate their current health on a 5-point scale using the question, “In general terms, how would you describe you rhealth: very good, good, fair, poor, or very poor?” A small number of participants were in extreme categories. Therefore, as suggested in several previous studies,[22, 23] we collapsed response options into 3 categories. These 3 categories were very good/good, fair, and poor/very poor.

The questionnaire included screening items for neurological disorders (dementia, cerebrovascular disease, Parkinson disease, and essential tremor). A short form of the questionnaire was mailed to participants who refused or were unavailable for face-to-face interview. The screening questions for dementia included the Spanish adaptation of a cognitive test (a 37-item version of the Mini-Mental State Examination [37-MMSE])[17, 18, 24–27] and an 11-item version of the Pfeffer Functional Activities Questionnaire (FAQ).[28] The FAQ assesses common activities that require complex cognitive and social functioning.[28] The total score for the 11 items ranges from 0 (completely independent) to 33 (completely dependent).[28] Those participants who screened positive for dementia underwent a neurological examination at National Health Service clinics or at home. The neurological assessment was comprised of a clinical history, a general neurological examination, and a cognitive and mental state examination. The neurological examination was performed by one of eight neurologists who met at the inception of the study to establish standardized methods to perform and interpret the examination (J. B-L, F. B-P., and see https://www.ciberned.es/en/estudio-nedices.html). For participants who could not be examined, medical records were obtained from their general practitioners, from in-patient hospitalizations, and from neurological specialists (if they had visited one). In addition to these medical records, death certificate diagnoses were reviewed for each screened participant who had died prior to their neurological examination.

The diagnosis of dementia was made by consensus of two neurologists based on clinical interview. For the diagnosis of dementia, we applied the Diagnostic and Statistical Manual of Mental Disorders (DSM)–IV criteria.[29]AD (possible or probable) was diagnosed according to NINCDS-ADRDA criteria.[30]

Follow-up data on death were collected until December 31, 2007. The date of death was obtained from the National Population Register of Spain (*Instituto Nacional de Estadística* in Spanish). In Spain, all deceased individuals receive a death certificate, completed by a physician, at the time of death. The certificate is then sent to the local authority in the municipality where the person had been living, and the information is added to the National Register. The cause of death (using the International Classification of Diseases—ICD- 9th Revision for deaths occurred prior to 1999, [http://www.cdc.gov/nchs/icd/icd9.htm], and the ICD 10th Revision [http://www.cdc.gov/nchs/icd/icd10.htm], for deaths occurring thereafter) was revised and classified by NEDICES researchers into one of six primary categories: AD, other causes of dementia, cerebrovascular disorders, cardiovascular disorders, respiratory diseases, cancer, and other causes (infections, trauma, genitourinary or gastrointestinal disorders). In agreement with the World Health Organization, the classification of causes of death had been codified by the physicians who completed the death certificates, depending on the basic cause of death (http://www.who.int/topics/mortality/en/). This was defined as the illness or injury that started the chain of pathological events, which directly led to death (http://www.who.int/topics/mortality/en/).

### Final selection of participants

**Fig 1:**
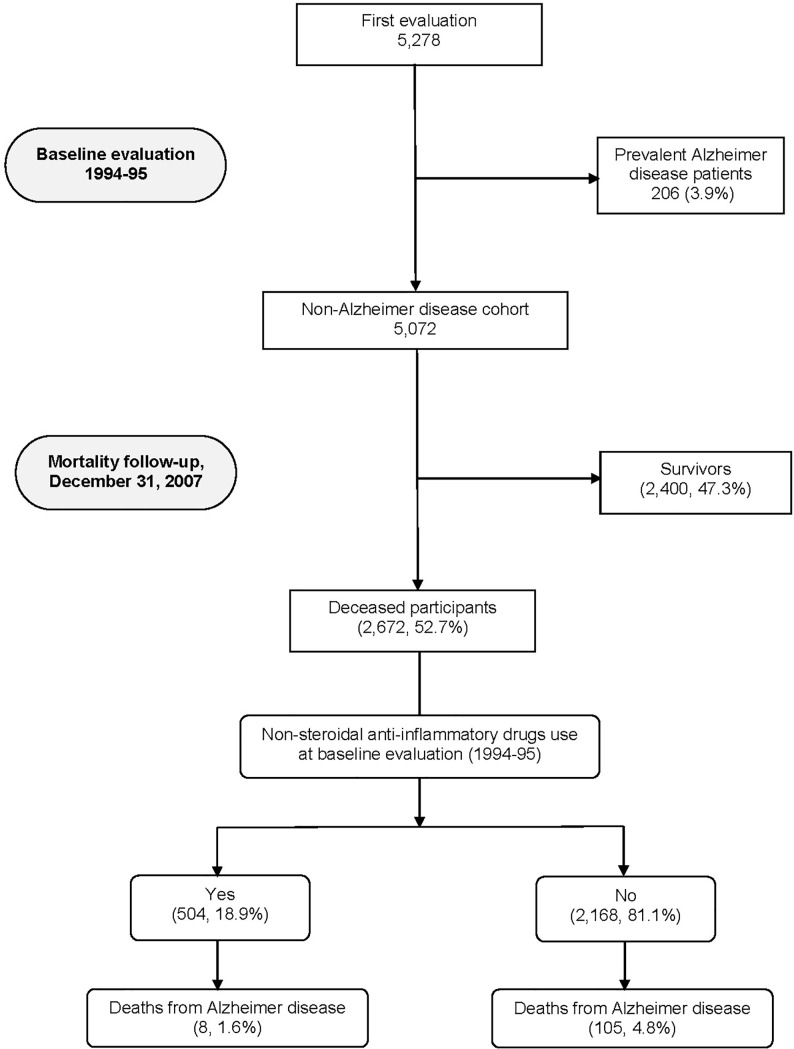
Flow chart of the study.

Of the 5,278 participants evaluated at baseline, we excluded 206 with AD diagnosed or detected by NEDICES researchers at baseline evaluation (1994–1995),[17] which left 5,072 remaining participants who were included in our analyses (Fig 1).

### Statistical analyses

Analyses were performed in SPSS (version 25.0). All tests were two sided, and significance was accepted at the 5% level (α = 0.05). Using a one-sample Kolmogorov–Smirnov test, we determined that age, FAQ total score, and a comorbidity index that included diabetes mellitus, hyperlipidemia, heart disease, cancer, anemia, chronic obstructive pulmonary disease, psychiatric disorders, osteoarthritis, osteoporosis, hypoacusis, cataracts, and peripheral vascular disease, were not normally distributed, even after log-transformation. Therefore, although mean and median values were reported, differences were compared using a nonparametric (Mann–Whitney U and Kruskal Wallis tests). The chi-square or Fisher p tests were used to analyze categorical variables. Participants were divided in NSAIDs users and non-users (reference category).

We used Cox proportional-hazards models to estimate hazard ratios (HRs) for AD mortality; this also generated 95% confidence intervals (CIs). The time variable was the years from the date of the first evaluation (1994 to 1995) to the date of death. The dependent (outcome) variable was the presence of AD as a cause of death, with the remaining causes of death serving as the reference group; meanwhile the independent (exposure) variable was the NSAIDs use category at baseline (NSAIDs use vs. non-use [reference category]). We began with an unadjusted model. Then, in adjusted models, we considered baseline variables that in bivariate analyses were associated at the p < 0.05 level with either the exposure or the outcome (“Model 1). Finally, for completeness, we adjusted for all the potential confounders, independent of their statistical significance (i.e., even if they were not associated with either the exposure or the outcome) (“Model 2”). Age in years, sex, educational level (illiterate, can read and write, primary studies, secondary and higher studies), living area during childhood/adolescence (rural vs urban area), marital status (married or domestic partnership, single, separated or divorced, and widowed), self-rated health (good/very good, fair, and bad/very bad), smoker (ever vs never), drinker (ever vs never), FAQ total score, the comorbidity index, arterial hypertension (drug-untreated hypertension, drug-treated hypertension, and no hypertension), and cerebrovascular disease (stroke and transient ischemic attack) were assessed at baseline and considered as potential covariates.

## Results

The 5,072 participants had a mean duration of follow-up of 10.0 years (median = 12.7 years; range = 0.01–14.2 years). Of the 5,072 participants, 2,672 (52.7%) died over a median follow-up of 6.8 years (range 0.01–13.8 years), including 504 (18.9%) NSAIDs users and 2,168 (81.1%) non-users. Of the 2,672 deceased participants, 113 (4.2%) had AD as a cause of death (8 [1.6%] among NSAIDs users and 105 [4.8%] among non-users, chi-square = 10.70, p = 0.001) (Fig 1). Table 1 shows the different NSAIDs types which were taking the 504 participants.

**Table 1:** Non-steroidal anti-inflammatory drugs types.

Non-steroidal anti-inflammatory drugs types	Number of participants (%)
Aspirin	322 (63.9%)
Diclofenac	42 (8.3%)
Piroxicam	24 (4.8%)
Aceclofenac	20 (4.0%)
Tenoxicam	12 (2.4%)
Indomethacin	19 (3.8%)
Naproxen	9 (1.8%)
Nabumetone	7 (1.4%)
Flurbiprofen	5 (1.0%)
Ibuprofen	4 (0.8%)
Ketoprofen	4 (0.8%)
Ketorolac	3 (0.6%)
Droxicam	2 (0.4%)
Meloxicam	1 (0.2%)
Sulindac	1 (0.2%)
Niflumic acid	1 (0.2%)
Acemetacin	1 (0.2%)
Isonixin	1 (0.2%)
Aspirin and diclofenac	8 (1.6%)
Aspirin and aceclofenac	3 (0.6%)
Aspirin and indomethacin	2 (0.4%)
Aspirin and ketoprofen	2 (0.4%)
Aspirin and tenoxicam	2 (0.4%)
Aspirin and piroxicam	2 (0.4%)
Aspirin and ibuprofen	1 (0.2%)
Aspirin and nabumetone	1 (0.2%)
Aspirin and sulindac	1 (0.2%)
Aspirin and naproxen	1 (0.2%)
Aspirin and droxicam	1 (0.2%)
Aspirin and mefenamic acid	1 (0.2%)
Aspirin and isonixin	1 (0.2%)

Baseline demographic and clinical characteristics of deceased participants who were taking NSAIDs and participants who were not are shown in Table 2. A higher proportion of NSAIDs users were more educated. In addition, NSAIDs users were more likely to have higher FAQ total scores, more drug-treated arterial hypertension, and more cerebrovascular diseases.

**Table 2:** Baseline (1994–1995) demographic and clinical characteristics of deceased participants (N = 2,672) who were taking non-steroidal anti-inflammatory drugs and participants who were not.

	Participants taking on-steroidal anti-inflammatory drugs (N = 504)	Participants who were not taking non-steroidal anti-inflammatory drugs (N = 2,168)	*p* value
Age in years	76.7 (76.5) ± 6.7	76.4 (76.0) ± 7.1	0.341^a^
Sex (women)	237 (47.0%)	1,088 (50.2%)	0.201^b^
Educational level *			0.047^b^
*Illiterate*	63 (12.7%)	298 (13.9%)	
*Can read and write*	212 (42.7%)	840 (39.2%)	
*Primary studies*	138 (27.8%)	713 (33.2%)	
*Secondary and higher studies*	83 (16.7%)	294 (13.7%)	
Living area during childhood/adolescence *			0.603^b^
*Rural*	136 (33.5%)	514 (32.1%)	
*Urban*	270 (66.5%)	1,085 (67.9%)	
Marital Status *			0.169^b^
*Married or domestic partnership*	222 (54.1%)	947 (57.6%)	
*Single*	30 (7.3%)	153 (9.3%)	
*Separated or divorced*	7 (1.7%)	26(1.6%)	
*Widowed*	151 (36.8%)	518 (31.5%)	
Self-rated health *			0.113^b^
*Good / very good*	228 (48.2%)	1,032 (52.0%)	
*Fair*	155 (32.8%)	649 (32.7%)	
*Bad / very bad*	90 (19.0%)	304 (15.3%)	
Ever smoker (ex-smoker plus current smoker) *	168 (44.7%)	593 (39.2%)	0.052^b^
Ever drinker (ex-drinker plus current drinker) *	239 (59.0%)	935 (57.9%)	0.674^b^
FAQ total score *	4.3 (1.0) ± 6.8	3.4 (0.0) ± 6.3	0.004^a^
Comorbidity index* #	2.7 (3.0) ± 1.8	2.6 (2.0) ± 1.7	0.063^a^
Arterial hypertension *			< 0.001 ^b^
*Drug-untreated hypertension*	35 (7.2%)	160 (7.9%)	
*Drug-treated hypertension*	283 (58.6%)	917 (45.3%)	
*No hypertension*	165 (34.2%)	947 (46.8%)	
Cerebrovascular disease (stroke and transient ischemic attack)	70 (13.9%)	115 (5.3%)	< 0.001 ^b^

^a^ Mann-Whitney test

^b^ Chi-square test. Mean (median) ± standard deviation and frequency (%) are reported.

* Data on some participants were missing.

# Comorbidity included 12 conditions: diabetes mellitus, hyperlipidemia, heart diseases, cancer, anemia, chronic obstructive pulmonary disease, psychiatric disorders, osteoarthritis, osteoporosis, hypoacusis, cataracts, and peripheral vascular disease. FAQ: Pfeffer Functional Activities Questionnaire.

Baseline characteristics of the participants who died from AD vs. those who died from other causes are shown in Table 3. Those who died from AD were more likely to have been less smokers. In addition, they were more likely to have less comorbidities, but more untreated arterial hypertension.

**Table 3:** Baseline (1994–1995) demographic and clinical characteristics of participants who died from Alzheimer’s disease vs other causes.

	Participants who died from Alzheimer disease (N = 113)	Participants who died from other causes (N = 2,559)	*p* value
Age in years	77.4 (76.0) ± 6.8	76.4 (76.0) ± 7.0	0.154^a^
Sex (women)	64 (56.6%)	1,261 (49.3%)	0.126^b^
Educational level *			0.416^b^
*Illiterate*	12 (10.6%)	349 (13.8%)	
*Can read and write*	53 (46.9%)	999 (39.5%)	
*Primary studies*	32 (28.3%)	819 (32.3%)	
*Secondary and higher studies*	16 (14.2%)	361 (14.3%)	
Living area during childhood/adolescence *			0.648^b^
*Rural*	25 (30.1%)	625 (32.5%)	
*Urban*	58 (69.9%)	1,297 (67.5%)	
Marital Status *			0.228^c^
*Married or domestic partnership*	44 (52.4%)	1,125 (57.1%)	
*Single*	4 (4.8%)	179 (9.1%)	
*Separated or divorced*	1 (1.2%)	32 (1.6%)	
*Widowed*	35 (41.7%)	634 (32.2%)	
Self-rated health *			0.320^b^
*Good / very good*	59 (57.8%)	1,201 (51.0%)	
*Fair*	31 (30.4%)	773 (32.8%)	
*Bad / very bad*	12 (11.8%)	382 (16.2%)	
Ever smoker (ex-smoker plus current smoker) *	25 (29.4%)	736 (40.8%)	0.036^b^
Ever drinker (ex-drinker plus current drinker) *	42 (48.8%)	1,132 (58.5%)	0.076^b^
Pfeffer Functional Activities Questionnaire total score *	2.8 (0.0) ± 5.6	3.6 (0.0) ± 6.4	0.644^a^
Comorbidity index*#	2.1 (2.0) ± 1.6	2.6 (2.0) ± 1.7	<0.001^a^
Arterial hypertension *			< 0.001 ^b^
*Drug-untreated hypertension*	18 (16.8%)	177 (7.4%)	
*Drug-treated hypertension*	34 (31.8%)	1,166 (48.6%)	
*No hypertension*	55 (51.4%)	1,057 (44.0%)	
Cerebrovascular disease (stroke and transient ischemic attack)	4 (3.5%)	181 (7.1%)	0.148^b^

^a^ Mann-Whitney test

^b^ Chi-square test.

^c^Fisher p test. Mean (median) ± standard deviation and frequency (%) are reported.

* Data on some participants were missing.

# Comorbidity included 12 conditions: diabetes mellitus, hyperlipidemia, heart diseases, cancer, anemia, chronic obstructive pulmonary disease, psychiatric disorders, osteoarthritis, osteoporosis, hypoacusis, cataracts, and peripheral vascular disease.

In an unadjusted Cox model, risk of AD mortality was decreased in NSAIDs users vs. non-users (Table 4). In a Cox model that adjusted for variables associated with either NSAIDs use or AD mortality (i.e., baseline educational level, smoker, FAQ total score, comorbidity index, arterial hypertension, and cerebrovascular disease), the risk of mortality remained decreased in NSAIDs users (Model 1, Table 4). This effect remained significant after adjusting the model for baseline age, sex, educational level, living area during childhood/adolescence, marital status, self-rated health, smoker, drinker, FAQ total score, comorbidity index, arterial hypertension, and cerebrovascular disease (i.e., all potential confounders independent of their statistical significance) (Model 2, Table 4).

**Table 4:** Risks of Alzheimer’s disease mortality in deceased participants (N = 2,672) who were taking non-steroidal anti-inflammatory drugs and participants who were not (reference group).

	Unadjusted			Model 1			Model 2		
	Hazard ratio	95% CI	*p* value	Hazard ratio	95% CI	*p* value	Hazard ratio	95% CI	*p* value
Non-steroidal anti-inflammatory drugs users N = 504	0.35	0.17–0.72	0.004	0.29	0.12–0.72	0.008	0.29	0.12–0.73	0.009
Participants who were not taking non-steroidal anti-inflammatory drugs N = 2,168 (reference category)	1.00	_		1.00	_		1.00	_	

Model 1: Adjusted for baseline educational level, smoker, Pfeffer Functional Activities Questionnaire total score, comorbidity index, arterial hypertension, and cerebrovascular disease, chi-square = 21.78, p = 0.003.

Model 2: Adjusted for baseline age, sex, educational level, living area during childhood/adolescence, marital status, self-rated health, smoker, drinker, Pfeffer Functional Activities Questionnaire total score, comorbidity index, arterial hypertension, and cerebrovascular disease, chi-square = 29.10, p = 0.006.

We also conducted a sensitivity analysis in which we excluded all participants (N = 156) who were taking only non-aspirin NSAIDs. In these analyses, the HRs for AD mortality in aspirin users remained decreased (HR = 0.42, 95% CI = 0.18–0.95, p = 0.037, unadjusted Cox model; HR = 0.27, 95% CI = 0.10–0.90, p = 0.033, Model 1; and HR = 0.28, 95% CI = 0.10–0.90, p = 0.033, Model 2). In another analysis we excluded the 322 participants who were taking only aspirin. In these analyses, the HRs for AD mortality in non-aspirin NSAIDs users remained decreased but did not reach statistical significance in adjusted Cox models (HR = 0.30, 95% CI = 0.10–0.94, p = 0.039, unadjusted Cox model; HR = 0.26, 95% CI = 0.10–1.10, p = 0.060, Model 1; and HR = 0.25, 95% CI = 0.10–1.03, p = 0.056, Model 2).

In a final analysis, we analyzed the risk of AD mortality in those taking acetaminophen (N = 153) vs. those who were not. In these analyses, the risk of AD mortality in acetaminophen users was not significant (HR = 1.05, 95% CI = 0.46–2.38, p = 0.916, unadjusted Cox model; HR = 0.83, 95% CI = 0.30–2.30, p = 0.722, Model 1; and HR = 0.73, 95% CI = 0.22–2.34, p = 0.593, Model 2).

## Discussion

The results of the current study suggest that non-AD older adults taking NSAIDs are at decreased risk of AD mortality. Relative to non-users, the HR for AD as underlying cause of death was 71% lower in NSAIDs users. The association persisted even after controlling for a variety of combinations of covariates in adjusted models. Stratified analyses by individual NSAID type showed a significantly decreased risk of AD mortality with aspirin, whereas non-aspirin NSAIDs only showed a statistical trend toward significance in the adjusted Cox regression models. The fact that the non-aspirin NSAID-AD mortality association was not statistically significant may be due to small numbers, rather than to the lack of a real association.

It remains to be established how the relation between NSAIDs, particularly with aspirin, and AD mortality is mediated. We can speculate on possible explanations for this association. First, the inhibition of the cyclooxygenase isoenzymes (1 and 2) by NSAIDs reduces the levels of substances that are known to be related with AD pathogenesis, such as prostaglandins, prostacyclin, and thromboxanes,[31–33] Second, aspirin is an irreversible inhibitor of both cyclooxygenase-1 and cyclooxygenase-2, which is known to reduce oxidative stress and protect against oxidative damage.[34] In transgenic AD mice, selective cyclooxygenase-1 inhibition has been demonstrated to reduce neuroinflammation, amyloid pathology, and improvement of cognitive function.[35] Third, biomarker studies have shown that amyloid-β protein deposits in brain precedes AD onset more than a decade before cognitive deficits appear.[36, 37] It has been hypothesized that NSAIDs use may be beneficial only in the normal brain by inhibiting the production of amyloid-β protein.[38] Once the abnormal deposition process of amyloid-β protein has started, NSAIDs are no longer effective and may even be detrimental because of their inhibiting activity on activated microglia of the AD brain, which mediates amyloid-β protein clearance and activates compensatory hippocampal neurogenesis.[38] This would explain, on one hand, why epidemiological studies suggest that NSAIDs can ameliorate this neurodegeneration process if they are started before clinical signs develop and, on the other, the disappointing results of clinical trials in AD patients.[39]

Our study has limitations. First, we did not consider the dose and frequency of NSAIDs use, so we were not able to evaluate whether higher current dose or cumulative dose of these medications was associated with lower risk of AD mortality. Second, data on NSAIDs exposure were available at baseline, but not for any intermediate time intervals between baseline and follow-up. Such data would have been of value in terms of assessing variation in drug exposure over time. Third, we based the diagnoses of AD on the revision of death certificates. AD may be omitted as a cause of death from the death certificates of patients with known AD in life.[40] Such omissions, however, are equally likely for NSAID users and non-users. Fourth, the residual confounding distortion by unmeasured factors is possible, but a large range of covariates were considered for Cox proportional hazards analyses. Finally, competing mortality is a potential issue in this type of studies. Indeed, there could be a potential for survival bias, considering that a small proportion of individuals may have died from AD at younger ages. However, AD is an ageing-related disease and competing mortality could have a null or limited influence on our findings.

Despite these limitations, the study was population-based, allowing us to assess a group of participants who were unselected for treatment considerations. In addition, NSAIDs users were compared to a large sample size of several hundred NSAIDs non-users. Finally, we could adjust for the potential confounding effects of several crucial factors.

In NEDICES, we demonstrated that NSAIDs use was associated with decreased risk of AD mortality, even after controlling for confounders. Our results support the hypothesis that NSAIDs use is a protective factor of developing AD.

## References

[1] Ali MM, Ghouri RG, Ans AH, Akbar A, Toheed A. Recommendations for Anti-inflammatory Treatments in Alzheimer's Disease: A Comprehensive Review of the Literature. Cureus. 2019;11(5):e4620 Epub 2019/07/18. 10.7759/cureus.4620 31312547 PMC6615583

[2] McGeer PL, McGeer E, Rogers J, Sibley J. Anti-inflammatory drugs and Alzheimer disease. Lancet (London, England). 1990;335(8696):1037 Epub 1990/04/28. 10.1016/0140-6736(90)91101-f . 1970087

[3] Wang J, Tan L, Wang HF, Tan CC, Meng XF, Wang C, et al Anti-inflammatory drugs and risk of Alzheimer's disease: an updated systematic review and meta-analysis. Journal of Alzheimer's disease: JAD. 2015;44(2):385–96. 10.3233/JAD-141506 . 25227314

[4] Jaturapatporn D, Isaac MG, McCleery J, Tabet N. Aspirin, steroidal and non-steroidal anti-inflammatory drugs for the treatment of Alzheimer's disease. Cochrane Database Syst Rev. 2012;(2):CD006378 10.1002/14651858.CD006378.pub2 . 22336816 PMC11337172

[5] Meyer PF, Tremblay-Mercier J, Leoutsakos J, Madjar C, Lafaille-Maignan ME, Savard M, et al INTREPAD: A randomized trial of naproxen to slow progress of presymptomatic Alzheimer disease. Neurology. 2019;92(18):e2070–e80. Epub 2019/04/07. 10.1212/WNL.0000000000007232 PubMed Central PMCID: PMC6512884. 30952794 PMC6512884

[6] Nevado-Holgado AJ, Lovestone S. Determining the Molecular Pathways Underlying the Protective Effect of Non-Steroidal Anti-Inflammatory Drugs for Alzheimer's Disease: A Bioinformatics Approach. Comput Struct Biotechnol J. 2017;15:1–7. 10.1016/j.csbj.2016.10.003 27872687 PMC5109283

[7] Sanz-Blasco S, Calvo-Rodríguez M, Caballero E, García-Durillo M, Nuñez L, Villalobos C. Is it All Said for NSAIDs in Alzheimer's Disease? Role of Mitochondrial Calcium Uptake. Curr Alzheimer Res. 2018;15(6):504–10. 10.2174/1567205015666171227154016 . 29283047

[8] Hoyer S. Oxidative metabolism deficiencies in brains of patients with Alzheimer's disease. Acta neurologica Scandinavica Supplementum. 1996;165:18–24. . 8740985 10.1111/j.1600-0404.1996.tb05868.x

[9] Benito-León J, Louis ED, Villarejo-Galende A, Labiano-Fontcuberta A, Bermejo-Pareja F. Self-rated health and risk of incident essential tremor: A prospective, population-based study (NEDICES). Parkinsonism Relat Disord. 2015;21(6):622–8. Epub 2015/04/19. 10.1016/j.parkreldis.2015.03.023 . 25887487

[10] Zhang C, Wang Y, Wang D, Zhang J, Zhang F. NSAID Exposure and Risk of Alzheimer's Disease: An Updated Meta-Analysis From Cohort Studies. Frontiers in Aging Neuroscience. 2018;10(83). 10.3389/fnagi.2018.00083 29643804 PMC5882872

[11] Varga Z, Sabzwari SRA, Vargova V. Cardiovascular Risk of Nonsteroidal Anti-Inflammatory Drugs: An Under-Recognized Public Health Issue. Cureus. 2017;9(4):e1144 10.7759/cureus.1144 28491485 PMC5422108

[12] Bermejo-Pareja F, Benito-León J, Louis ED, Trincado R, Carro E, Villarejo A, et al Risk of incident dementia in drug-untreated arterial hypertension: a population-based study. Journal of Alzheimer's disease: JAD. 2010;22(3):949–58. Epub 2010/09/23. 10.3233/JAD-2010-101110 . 20858957

[13] Benito-León J, Bermejo-Pareja F, Morales JM, Vega S, Molina JA. Prevalence of essential tremor in three elderly populations of central Spain. Mov Disord. 2003;18(4):389–94. Epub 2003/04/03. 10.1002/mds.10376 . 12671944

[14] Benito-León J, Bermejo-Pareja F, Rodriguez J, Molina JA, Gabriel R, Morales JM, et al Prevalence of PD and other types of parkinsonism in three elderly populations of central Spain. Mov Disord. 2003;18(3):267–74. Epub 2003/03/07. 10.1002/mds.10362 . 12621629

[15] Benito-León J, Bermejo-Pareja F, Morales-González JM, Porta-Etessam J, Trincado R, Vega S, et al Incidence of Parkinson disease and parkinsonism in three elderly populations of central Spain. Neurology. 2004;62(5):734–41. Epub 2004/03/10. 10.1212/01.wnl.0000113727.73153.68 . 15007123

[16] Díaz-Guzmán J, Bermejo-Pareja F, Benito-León J, Vega S, Gabriel R, Medrano MJ, et al Prevalence of stroke and transient ischemic attack in three elderly populations of central Spain. Neuroepidemiology. 2008;30(4):247–53. Epub 2008/06/03. 10.1159/000135643 . 18515974

[17] Bermejo-Pareja F, Benito-León J, Vega S, Olazarán J, de Toledo M, Díaz-Guzmán J, et al Consistency of clinical diagnosis of dementia in NEDICES: A population-based longitudinal study in Spain. J Geriatr Psychiatry Neurol. 2009;22(4):246–55. Epub 2009/05/07. 10.1177/0891988709335794 . 19417217

[18] Bermejo-Pareja F, Benito-León J, Vega S, Medrano MJ, Roman GC, Neurological Disorders in Central Spain Study G. Incidence and subtypes of dementia in three elderly populations of central Spain. J Neurol Sci. 2008;264(1–2):63–72. Epub 2007/08/31. 10.1016/j.jns.2007.07.021 . 17727890

[19] Morales JM, Bermejo FP, Benito-León J, Rivera-Navarro J, Trincado R, Gabriel SR, et al Methods and demographic findings of the baseline survey of the NEDICES cohort: a door-to-door survey of neurological disorders in three communities from Central Spain. Public Health. 2004;118(6):426–33. 10.1016/j.puhe.2003.10.007 15313596

[20] Bermejo-Pareja F, Benito-León J, Vega QS, Díaz-Guzmán J, Rivera-Navarro J, Molina JA, et al [The NEDICES cohort of the elderly. Methodology and main neurological findings]. Rev Neurol. 2008;46(7):416–23. Epub 2008/04/05. . 18389461

[21] Vega S, Benito-León J, Bermejo-Pareja F, Medrano MJ, Vega-Valderrama LM, Rodríguez C, et al Several factors influenced attrition in a population-based elderly cohort: neurological disorders in Central Spain Study. J Clin Epidemiol. 2010;63(2):215–22. Epub 2009/05/29. 10.1016/j.jclinepi.2009.03.005 . 19473811

[22] Benito-León J, Contador I, Louis ED, Cosentino S, Bermejo-Pareja F. Education and risk of incident dementia during the premotor and motor phases of essential tremor (NEDICES). Medicine (Baltimore). 2016;95(33):e4607 10.1097/MD.0000000000004607 . 27537597 PMC5370823

[23] Benito-León J, Romero JP, Louis ED, Bermejo-Pareja F. Faster cognitive decline in elders without dementia and decreased risk of cancer mortality: NEDICES Study. Neurology. 2014;82(16):1441–8. Epub 2014/04/11. 10.1212/WNL.0000000000000350 24719490 PMC4001199

[24] Benito-León J, Louis ED, Bermejo-Pareja F, Neurological Disorders in Central Spain Study G. Population-based case-control study of cognitive function in essential tremor. Neurology. 2006;66(1):69–74. Epub 2006/01/13. 10.1212/01.wnl.0000192393.05850.ec . 16401849

[25] Benito-León J, Louis ED, Vega S, Bermejo-Pareja F. Statins and cognitive functioning in the elderly: a population-based study. Journal of Alzheimer's disease: JAD. 2010;21(1):95–102. Epub 2010/04/24. 10.3233/JAD-2010-100180 . 20413854

[26] Benito-León J, Mitchell AJ, Vega S, Bermejo-Pareja F. A population-based study of cognitive function in older people with subjective memory complaints. Journal of Alzheimer's disease: JAD. 2010;22(1):159–70. 10.3233/JAD-2010-100972 20847410

[27] Benito-León J, Louis ED, Sánchez-Ferro A, Bermejo-Pareja F. Rate of cognitive decline during the premotor phase of essential tremor: a prospective study. Neurology. 2013;81(1):60–6. Epub 2013/05/24. 10.1212/WNL.0b013e318297ef2b 23700331 PMC3770204

[28] Louis ED, Benito-León J, Vega-Quiroga S, Bermejo-Pareja F, Neurological Disorders in Central Spain Study G. Cognitive and motor functional activity in non-demented community-dwelling essential tremor cases. J Neurol Neurosurg Psychiatry. 2010;81(9):997–1001. Epub 2010/06/16. 10.1136/jnnp.2009.202838 . 20547612

[29] American Psychiatric A. Diagnostic and Statistical Manual of Mental Disorders DSM-IV. Washington1994 1994.

[30] McKhann G, Drachman D, Folstein M, Katzman R, Price D, Stadlan EM. Clinical diagnosis of Alzheimer's disease: report of the NINCDS-ADRDA Work Group under the auspices of Department of Health and Human Services Task Force on Alzheimer's Disease. Neurology. 1984;34(7):939–44. 10.1212/wnl.34.7.939 6610841

[31] Kotilinek LA, Westerman MA, Wang Q, Panizzon K, Lim GP, Simonyi A, et al Cyclooxygenase-2 inhibition improves amyloid-beta-mediated suppression of memory and synaptic plasticity. Brain. 2008;131(Pt 3):651–64. Epub 2008/02/23. 10.1093/brain/awn008 18292081 PMC2628581

[32] Choi SH, Bosetti F. Cyclooxygenase-1 null mice show reduced neuroinflammation in response to beta-amyloid. Aging. 2009;1(2):234–44. Epub 2010/02/17. 10.18632/aging.100021 20157512 PMC2806008

[33] Woodling NS, Andreasson KI. Untangling the Web: Toxic and Protective Effects of Neuroinflammation and PGE2 Signaling in Alzheimer's Disease. ACS Chem Neurosci. 2016;7(4):454–63. Epub 2016/03/17. 10.1021/acschemneuro.6b00016 26979823 PMC5239037

[34] Hankey GJ, Eikelboom JW. Aspirin resistance. Lancet (London, England). 2006;367(9510):606–17. 10.1016/S0140-6736(06)68040-9 . 16488805

[35] Choi SH, Aid S, Caracciolo L, Minami SS, Niikura T, Matsuoka Y, et al Cyclooxygenase-1 inhibition reduces amyloid pathology and improves memory deficits in a mouse model of Alzheimer's disease. Journal of neurochemistry. 2013;124(1):59–68. Epub 2012/10/23. 10.1111/jnc.12059 23083210 PMC3780364

[36] Prestia A, Caroli A, van der Flier WM, Ossenkoppele R, Van Berckel B, Barkhof F, et al Prediction of dementia in MCI patients based on core diagnostic markers for Alzheimer disease. Neurology. 2013;80(11):1048–56. 10.1212/WNL.0b013e3182872830 . 23390179

[37] Buchhave P, Minthon L, Zetterberg H, Wallin AK, Blennow K, Hansson O. Cerebrospinal fluid levels of beta-amyloid 1–42, but not of tau, are fully changed already 5 to 10 years before the onset of Alzheimer dementia. Archives of general psychiatry. 2012;69(1):98–106. Epub 2012/01/04. 10.1001/archgenpsychiatry.2011.155 . 22213792

[38] Imbimbo BP. An update on the efficacy of non-steroidal anti-inflammatory drugs in Alzheimer's disease. Expert Opin Investig Drugs. 2009;18(8):1147–68. 10.1517/13543780903066780 . 19589092

[39] McGeer PL, Guo JP, Lee M, Kennedy K, McGeer EG. Alzheimer's Disease Can Be Spared by Nonsteroidal Anti-Inflammatory Drugs. Journal of Alzheimer's disease: JAD. 2018;62(3):1219–22. Epub 2017/11/06. 10.3233/JAD-170706 29103042 PMC5870017

[40] Romero JP, Benito-León J, Louis ED, Pareja FB. Under Reporting of Dementia Deaths on Death Certificates: A Systematic Review of Population-based Cohort Studies. J Alzheimers Dis. 2014 Epub 2014/03/04. 10.3233/JAD-132765 . 24583403

